# Does early exposure to spoken and sign language affect reading fluency in deaf and hard-of-hearing adult signers?

**DOI:** 10.3389/fpsyg.2023.1145638

**Published:** 2023-09-20

**Authors:** Anastasia A. Ziubanova, Anna K. Laurinavichyute, Olga Parshina

**Affiliations:** ^1^Center for Language and Brain, HSE University, Moscow, Russia; ^2^Department of Linguistics, University of Potsdam, Potsdam, Germany; ^3^Psychology Department, Middlebury College, Middlebury, VT, United States

**Keywords:** reading fluency, deaf, hard-of-hearing, sign language, multimodal bilingualism, scanpaths, eye movements

## Abstract

**Introduction:**

Early linguistic background, and in particular, access to language, lays the foundation of future reading skills in deaf and hard-of-hearing signers. The current study aims to estimate the impact of two factors – early access to sign and/or spoken language – on reading fluency in deaf and hard-of-hearing adult Russian Sign Language speakers.

**Methods:**

In the eye-tracking experiment, 26 deaf and 14 hard-of-hearing native Russian Sign Language speakers read 144 sentences from the Russian Sentence Corpus. Analysis of global eye-movement trajectories (scanpaths) was used to identify clusters of typical reading trajectories. The role of early access to sign and spoken language as well as vocabulary size as predictors of the more fluent reading pattern was tested.

**Results:**

Hard-of-hearing signers with early access to sign language read more fluently than those who were exposed to sign language later in life or deaf signers without access to speech sounds. No association between early access to spoken language and reading fluency was found.

**Discussion:**

Our results suggest a unique advantage for the hard-of-hearing individuals from having early access to both sign and spoken language and support the existing claims that early exposure to sign language is beneficial not only for deaf but also for hard-of-hearing children.

## 1. Introduction

Although able to reach high reading proficiency, deaf readers are on average less skilled than hearing ones (Goldin-Meadow and Mayberry, 2001; Luckner et al., 2005; Kelly and Barac-Cikoja, 2007). Poorer reading in deaf individuals was initially attributed to spoken language phonology deficit (Hanson, 1989), but later research indicated that phonological activation is not necessary for proficient reading (Mayberry et al., 2011; Bélanger et al., 2012, 2013; Clark et al., 2016; Thierfelder et al., 2020; *cf.* Blythe et al. (2018) arguing for phonological recoding and Yan et al. (2015) as well as Yan et al. (2021) arguing for phonological preview benefit). More recently, reading skills in deaf people have been associated with different social integration background and educational methods, personal cognitive and social strengths (Marschark et al., 2015), exposure to written language (Tomasuolo et al., 2019), silent lipreading (Kyle et al., 2016), and, most importantly, early language development (Padden and Ramsey, 2000; Mayberry, 2007; Freel et al., 2011; Lederberg et al., 2013; Clark et al., 2016; Tomasuolo et al., 2019).

The foundation of early language development is access to language. In deaf and hard-of-hearing people, access to language can take different paths, be that access to sign language, to spoken language, or both. The precise role of each route for reading proficiency is under debate. Mayberry and Lock (2003; see also Clark et al., 2016) claim that it is early sign language acquisition that is essential for later reading abilities (based on data from children with severe and profound hearing loss, who have no access to the sounds of spoken language). Early acquisition of sign language is crucial not only for future proficiency in the sign language itself (in particular, for grammaticality judgments, Cormier et al., 2012; syntax, Boudreault and Mayberry, 2006; Henner et al., 2016; vocabulary, Caselli et al., 2021; Berger et al., 2023), but also for the later processing of written language (Clark et al., 2016). In particular, knowledge of American Sign Language syntax is correlated with the knowledge of English syntax (Chamberlain and Mayberry, 2008; Pinar et al., 2017; Hoffmeister et al., 2022); large vocabulary in Dutch Sign Language is correlated with large vocabulary in written Dutch (Hermans et al., 2008); better antonym knowledge in American Sign Language is correlated with better reading in English (Novogrodsky et al., 2014); better knowledge of American Sign Language is correlated with better comprehension of written English (Freel et al., 2011). Perhaps most convincingly, proficiency in American Sign Language was the single significant predictor of performance on nationally standardized measures of reading comprehension, English language use, and mathematics (Hrastinski and Wilbur, 2016).

However, early acquisition of sign language might be not the only road to proficient reading. Tomasuolo et al. (2019) found that deaf children of deaf parents, deaf oral monolinguals, and people with normal hearing had similar fixation durations during reading, and all outperformed deaf children of hearing parents who learned sign language only after the age of six. Tomasuolo and colleagues concluded that competence in either sign or spoken language is crucial for skilled reading in deaf. In a similar vein, Bertone and Volpato (2009) claim the critical role of (partial) access to spoken language: orally-trained children with access to speech sounds (cochlear implantation) outperformed all other groups of deaf children in a picture-matching task. To summarize, there is currently no consensus on whether it is access to sign or spoken language, or both that is important for future reading skills.

The first factor – early access to sign language – primarily depends on the hearing status of the child’s parents, since deaf parents tend to be signers, and hearing parents tend to either learn sign language together with their child (which might help children to gain age-appropriate SL vocabulary, see Berger et al., 2023) or opt for oral communication and education without any use of sign language. Deaf children born to deaf parents are likely to have early access to sign language and successfully acquire it as their first language. They are usually referred to as native signers, defined as having at least one deaf parent (here, we follow Tomasuolo et al., 2019; Hoffmeister et al., 2022, and others). In contrast, deaf children born to hearing parents may be deprived of sign language input – in fact, of any language input – as infants, which may hinder overall language development (Goldin-Meadow and Mayberry, 2001; Mayberry, 2007).

The second factor – early access to spoken language – depends on the degree of hearing loss of the child assuming other factors such as the quality of caretaker-child interactions, socioeconomic status, peer socialization, and cultural and individual differences are equal. For infants with some level of hearing loss, the severity of their hearing loss typically determines the amount of spoken language input they receive during infancy. Slight to moderately severe degrees of hearing loss correspond to the speech sound range the individuals perceive (see Table A2 in Appendix), and individuals with slight to moderate hearing loss have partial access to spoken language sounds. Hard-of-hearing children who have access to speech sounds from birth (e.g., from one or both parents, siblings, or other caretakers who use spoken language) are likely to acquire spoken language early. Children with severe and profound deafness are minimally exposed to spoken language sounds (only via lip-reading) and start learning spoken language later, already at school or at pre-school correction classes. Later exposure to spoken language may lead to lower spoken language proficiency (Bertone and Volpato, 2009).

The current study aims to add to the existing evidence on reading fluency in deaf and hard-of-hearing (DHH) signers: in addition to the early access to sign language, we also consider the access to spoken language approximated by the degree of hearing loss as a factor that can potentially influence reading fluency. While early access to sign language is clearly beneficial for reading skills of deaf individuals, it is less clear what role early access to spoken and/or sign language plays for hard-of-hearing individuals with partial access to speech sounds.

## 2. The present study

To investigate global reading fluency in DHH Russian signers, we focus not on the isolated measures related to individual word reading, such as fixation durations and skipping rates, but rather on the global trajectories of eye movements in reading the entire sentences (von der Malsburg and Vasishth, 2011). While the analysis of word-level eye movement characteristics is indispensable for studying how individual word properties affect reading, the analysis of scanpaths (i.e., sequences of eye movements) focuses on the bigger picture. Scanpath analysis combines fixation locations and their durations during reading the entire sentence into one continuous measure and allows the researchers to quantify the similarity between eye movement trajectories of different people.

**Figure 1:**
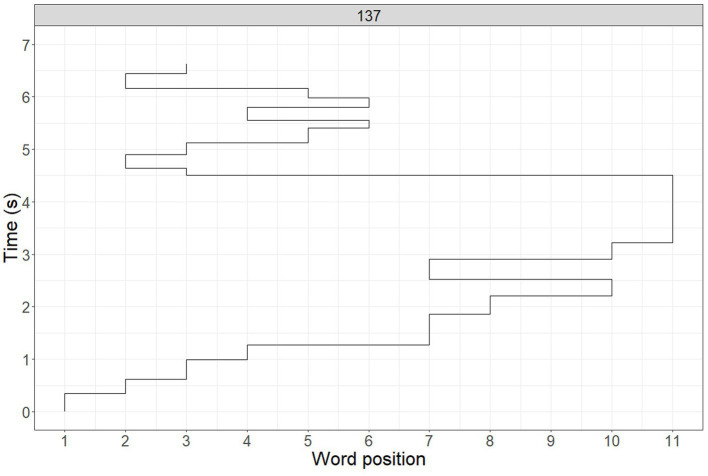
The example of gaze trajectory while sentence reading. The *y* axis shows sentence reading time in seconds and the *x* axis shows word position in the sentence.

To illustrate the concept of a scanpath, Figure 1 visualizes a trajectory of eye movements made while reading a sentence. The x-axis marks words in the sentence and the y-axis shows time in seconds. In this case, the reader fixated on the first word for about 400 ms and then continued to read the sentence word by word, skipped the 5th and the 6th words, fixated on the 7th and 8th words, skipped the 9th word and fixated on the 10th word, then made a regression to the 7th word, etc. This trajectory is an example of non-fluent reading: the scanpath includes six regressions and one atypically long fixation – the last word in the sentence was fixated for more than a second.

Utilizing the scanpath method to compare reading strategies in English-Russian bilingual and Russian-speaking monolingual speakers, Parshina et al. (2021a,b) found that monolingual adult readers followed the fluent reading strategy (fast sentence reading times, high word skipping rates, and almost no regressions), suggesting no difficulties in word recognition or syntactic and semantic information integration. Bilingual readers with early exposure to the second language and earlier exposure to the print language (e.g., heritage speakers of Russian who immigrated to the USA later in childhood) preferred the intermediate strategy (longer sentence reading times, lower word skipping rates, and more backward saccades to reread the words), indicative of delays in word recognition. Finally, bilingual readers with less exposure to spoken and written Russian (e.g., heritage speakers born in the USA) read according to the beginner strategy (even longer sentence reading times, more word and whole sentence rereadings), which the authors suggested reflects challenges not only in word recognition but also in the integration of morphosyntactic and semantic information.

In the present study (based on these findings and results in other studies, see above), we expect that early exposure to any type of language (sign or spoken) should be associated with greater reading fluency in DHH signers. That is, we expect higher reading fluency in both deaf and hard-of-hearing signers who have deaf parents and, therefore, were exposed to sign language from birth, and in hard-of-hearing signers who were exposed to spoken language from birth. We hypothesize that these readers will adopt a more fluent pattern of reading compared to DHH readers with less exposure to language (sign or spoken).

Admittedly, reading fluency *per se* is not a direct index of reading skill or successful comprehension: A text can be skimmed fast but poorly understood (Strukelj and Niehorster, 2018). Moreover, eye movements while reading depend not only on reading skill but also on reading goals and task demands (Mézière et al., 2021, 2022). For these reasons, we approximate reading skill through a combination of two measures: scanpaths, a combined measure capturing eye movements while reading, and questions probing sentence comprehension. A combination of skilled eye movement reading patterns and high question response accuracy would therefore index a better reading skill.

## 3. Methods

### 3.1. Participants

In Russia, deaf individuals are predominantly orally educated: they are taught to use monolingual spoken Russian as the primary means of production and lipreading for oral comprehension (Bazoev, 2016). This means that all DHH participants of the present study know spoken Russian and Russian print to some degree. Moreover, at the time of testing, all participants were daily users of Russian sign language (RSL; mean subjective assessment of proficiency = 8.97, SD = 1.46)^1^, which means that all participants were bilingual and bimodal in RSL, spoken Russian, and Russian print. Participants were recruited from the Head Educational, Research and Methodological Center for Vocational Rehabilitation of persons with disabilities at Bauman University in Moscow. All participants were compensated with 500 Rub. The study was approved by the HSE ethics committee.

The study included 40 DHH signers: 26 participants with complete hearing loss (*M*_age_ = 31, *SD* = 9) and 14 hard-of-hearing participants (*M*_age_ = 26, *SD* = 11). The individual characteristics of each participant can be found in Table A1 in Appendix. The group of deaf participants included people with severe and profound hearing loss. The hard-of-hearing group of participants included people whose level of hearing loss ranged from slight to moderately severe. The degree of hearing loss was self-reported based on the diagnosis by a medical practitioner (established on the basis of either otoacoustic emissions testing (OAE) or pure-tone audiometry).

Fifteen out of twenty-six deaf participants were born to deaf parents (recall that such individuals are considered to be native signers) and had hereditary deafness, while 11 were born in hearing families and had hearing loss due to other causes (see Table 1). Seven out of fourteen hard-of-hearing participants had deaf parents, the other seven had hearing parents. One participant from the hard-of-hearing group had a deaf mother and a hearing father and was classified as having deaf family due to access to sign language from birth.

**Table 1:** Characteristics of participants in each group.

	Deaf participants, hearing parents	Hard-of-hearing participants, hearing parents	Deaf participants, deaf parents	Hard-of-hearing participants, deaf parents	Stat. comparison
*Demographics*					
Total N	11	7	15	7	n.s.
Female participants	7	3	10	4	*
Vocabulary	33,272 (19,652)	52,571 (19,738)	51,466 (34,350)	49,571 (19,518)	n.s.
Age	28 (7.63)	25 (4.79)	33 (9.3)	27 (15.4)	n.s.
Start of RSL use	6.5 (3.1)	11.28 (5.49)	4 (1.4)	3.7 (2)	*
Years of education	16.54 (3.58)	16.42 (2.50)	17 (2.8)	13.6 (1.9)	n.s.
RSL proficiency (self-reported)	9.27 (1.55)	7.28 (1.49)	9.6 (0.8)	8.85 (1.2)	*
*Characteristics of reading*					
Accuracy	0.69 (0.46)	0.76 (0.43)	0.76 (0.43)	0.80 (0.40)	n.s.
Sentence reading times, ms	4,721 (1554)	4,692 (988)	4,611 (2117)	3,368 (979)	n.s.
Average fixation duration, ms	246 (128)	229 (118)	240 (122)	221 (106)	n.s.
Number of fixations on a sentence	19.2 (10)	20.5 (9.12)	19.2 (11)	15.3 (5.52)	n.s.

Statistical comparisons are based on the mixed-effects or linear models that the reader can find in the supplementary code. In the Statistical comparison column, n.s. stands for no significant differences. The significant differences between groups are as follows: Deaf participants and children of at least one deaf parent reported both earlier start of RSL use and higher RSL proficiency. In addition, there were significantly more females among deaf children of hearing parents than in other groups.

Individuals with at least one deaf parent are considered to be native signers. Numbers without parentheses represent counts or group means, numbers in parenthesis represent standard deviations. For the “Start of RSL use,” we encoded the starting age of those participants who said that they use RSL from childhood as 5 years, which is a conservative estimate.

The aim of the present study is to establish whether reading fluency of DHH signers is correlated with their parents’ hearing status and the individual degree of hearing loss. The mapping from these predictors to the main factors of interest, early access to sign and spoken language, is as follows: parents’ hearing status maps directly onto early access to sign language, which may benefit both deaf and hard-of-hearing individuals. In contrast, early access to spoken language maps onto the degree of participant’s individual hearing loss with more severe loss leading to lesser spoken input the individual receives during infancy. We also hypothesize that the degree of hearing loss might interact with the parents’ hearing status: the individuals with some access to speech sounds and at least one hearing parent are likely to have more early access to spoken sounds compared to individuals with deaf primary caretakers.

Materials. As reading materials, we used 144 sentences from the Russian Sentence Corpus developed as benchmark set of materials for assessing eye movements while reading in Russian (Laurinavichyute et al., 2019). The corpus is comprised of natural sentences randomly selected from the Russian National Corpus (https://Ruscorpora.ru) and normed for acceptability. Sentences had different syntactic structures: narratives, exclamations, and interrogatives, as well as sentences with non-standard word order. Sentences spanned from five to twelve words (with the average sentence length of 9 words) and were selected for being syntactically and lexically accessible. The Russian Sentence Corpus has been successfully read by advanced L2 learners and heritage speakers of Russian (Parshina et al., 2021b).

Originally, only 33% of the sentences in the corpus were followed by comprehension questions. To assess comprehension of DHH signers with higher precision, we introduced more questions: in the present study, 58% of sentences were followed by comprehension questions with three possible response options, see Example (1):

**Table:** 

(1)	Sentence	*Дорога ведет в глухой лес, петляя по склонам.* ‘The road leads into the deep forest, winding along the slopes.’
	Question	*Куда ведет дорога?* ‘Where does the road lead?’
	Correct answer	*В лес* ‘Into the forest’
	Incorrect answer 1	*В огород* ‘Into the garden’
	Incorrect answer 2	*В деревню* ‘Into the village.

In addition, approximate vocabulary size of print Russian was measured for each participant using an online computerized adaptive testing tool (Golovin, 2014; Andreev et al., 2016; Ashkinazi and Golovin, 2016). During the test, participants see a word or a non-word and have to indicate whether they know its meaning. If participants indicate that they know the meaning of the word, they may with some probability be asked to select a correct interpretation of the meaning or a correct synonym out of four options. If a participant knows infrequent words, even less frequent words are selected for further testing to estimate their vocabulary size with more precision.

### 3.2. Procedure

Stimuli were presented on the ASUS VG248QE monitor (resolution: 1,920 × 1,080 pix, response time: 1 ms, frame rate: 144 Hz, font face: 22-point Courier New). Eye movements were recorded at the rate of 1,000 Hz with desktop eye-tracker EyeLink 1,000+ using a chinrest. Eye-to-camera distance was 60 cm, eye-to-screen distance was 90 cm.

The experiment started with 9-dot camera calibration. After the calibration, a black dot was presented at the position of the first letter of the first word in the sentence. After the camera registered a fixation on the black dot, the sentence appeared. Participants were asked to read the sentence without signing (silent reading). If no fixation was registered on the black dot within 2 s, calibration was repeated. After having read the sentence, participants had to look at the red dot in the lower right corner of the screen. Fixation on the red dot triggered the next trial.

If the sentence was followed by a question, then after a fixation on the red dot was detected, the question appeared in place of the sentence. The response options were presented below the question. To select an answer, participants had to click on the response. The experiment started with three practice sentences and continued with 6 blocks, 24 experimental sentences in each. Between blocks, participants could have a break followed by a recalibration. The order in which the sentences appeared was randomized.

### 3.3. Analysis

To answer the main research question of the study, i.e., whether more proficient sentence reading trajectories in DHH participants are associated with early exposure to language, sign and/or spoken, we followed the steps in analysis in Parshina et al. (2021a,b). First, gaze trajectories (scanpaths) were recorded for all sentences for each participant. Trajectories with similar spatial and temporal characteristics (calculated using the Levenshtein distance) were then automatically grouped into clusters. To that end, we applied Gaussian mixture modeling (using the mclust package for R; Fraley and Raftery, 2007) that allowed us to identify the optimal number of clusters in each sentence. The advantage of using Gaussian mixture modeling over other clustering techniques (e.g., k-means clustering) is the method’s ability to detect clusters even in the presence of overlapping parameters. The median number of clusters for the entire corpus was 2 clusters, ranging from 1 to 9 clusters in each sentence. To facilitate interpretation and to avoid capturing random variation in reading patterns we proceeded to fit the models with the fixed number of 2 Gaussians for all sentences in the corpus. Any participant could read some sentences more fluently, and others more effortfully, so the same person’s reading trajectories for different sentences could be placed in different clusters. However, we expected that for each participant, one cluster would be dominant.

To find out whether early exposure to language affects cluster placement in DHH participants, we used a generalized mixed-effects model with the cluster as a dependent variable and parents’ hearing status, participant’s degree of hearing loss as predictors. We additionally used participants’ vocabulary size, age, and gender as covariates, as these factors are known to affect reading (Baumann, 2014; von der Malsburg et al., 2015; Reilly et al., 2019). The model also included the age at which participants started learning RSL, as RSL proficiency might play a role in reading (Hrastinski and Wilbur, 2016). The model was fit using `lme4` package (Bates et al., 2014), with dummy-coded categorical fixed effects (hearing parents coded as 0, deaf parents as 1; hard of hearing participants coded as 0, deaf participants as 1). Vocabulary size, age, and the age at which participants started learning RSL were centered and scaled; gender was coded as 1 for female, −1 for male participants. The random effects structure included random intercepts for participants and sentences, as well as by-sentence random slopes for the fixed effects of participants’ hearing status, their parents’ hearing status, and the interaction of these effects. Correlations between random slopes were not estimated.

The data and analysis code are openly available at: https://osf.io/je8du/. The readers are encouraged to reproduce our analysis and to apply any other analyses they see fit to the data set.

## 4. Results

**Figure 2:**
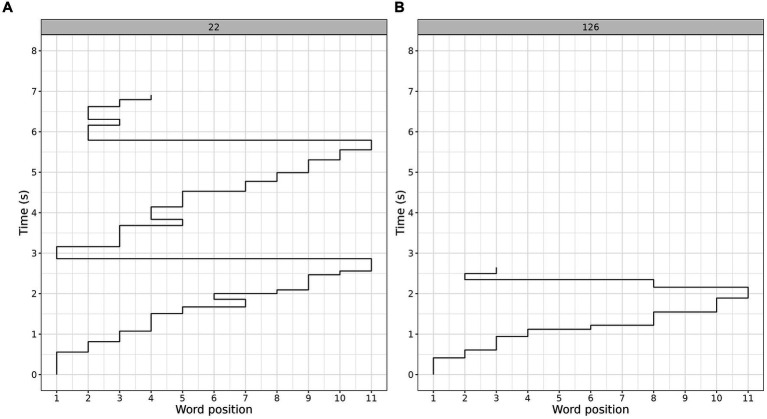
Gaze trajectories for reading the same sentence corresponding to typical reading patterns in the less-fluent **(A)** and more-fluent **(B)** clusters.

Based on the eye-movement characteristics, the two clusters earlier identified via Gaussian mixture modeling were labeled as more fluent and less fluent reading clusters (see Figure 2 for an example of typical gaze trajectories corresponding to the less-fluent and more-fluent reading clusters).

The less-fluent cluster was characterized by longer sentence reading times, longer fixation durations, greater number of fixations and regressions, and lower question response accuracy (see Table 2; note that in contrast to the eye-tracking measures, response accuracies were not used to compute the clusters). Both the less-fluent and the more-fluent reading clusters differed from the typical reading pattern of fluent monolingual Russian speakers reading the same materials [as reported in Parshina et al. (2021a,b)]. For comparison, monolingual Russian speakers had, on average, reading time of 2.1 s, and made 1.3 fixations per word (Parshina et al., 2021a,b).

**Table 2:** Comparison of eye-movement measures and question response accuracies in the less-fluent vs. more-fluent cluster.

	Less-fluent reading	More-fluent reading	*p*-value
Accuracy, *M* (*SD*)	0.69 (0.46)	0.79 (0.41)	**0.002**
Number of fixations/sentence, *M* (*SD*)	35 (15)	23 (10)	**<0.001**
Number of fixations/word, *M* (*SD*)	2.9 (2.2)	1.9 (1.4)	**<0.001**
Sentence reading time, *M* (*SD*), s	9.1 (4)	5.5 (3)	**<0.001**
Fixation duration*, *M* (*SD*), ms	260 (174)	245 (156)	**<0.001**

Bold values indicate statistical significance.

**Figure 3:**
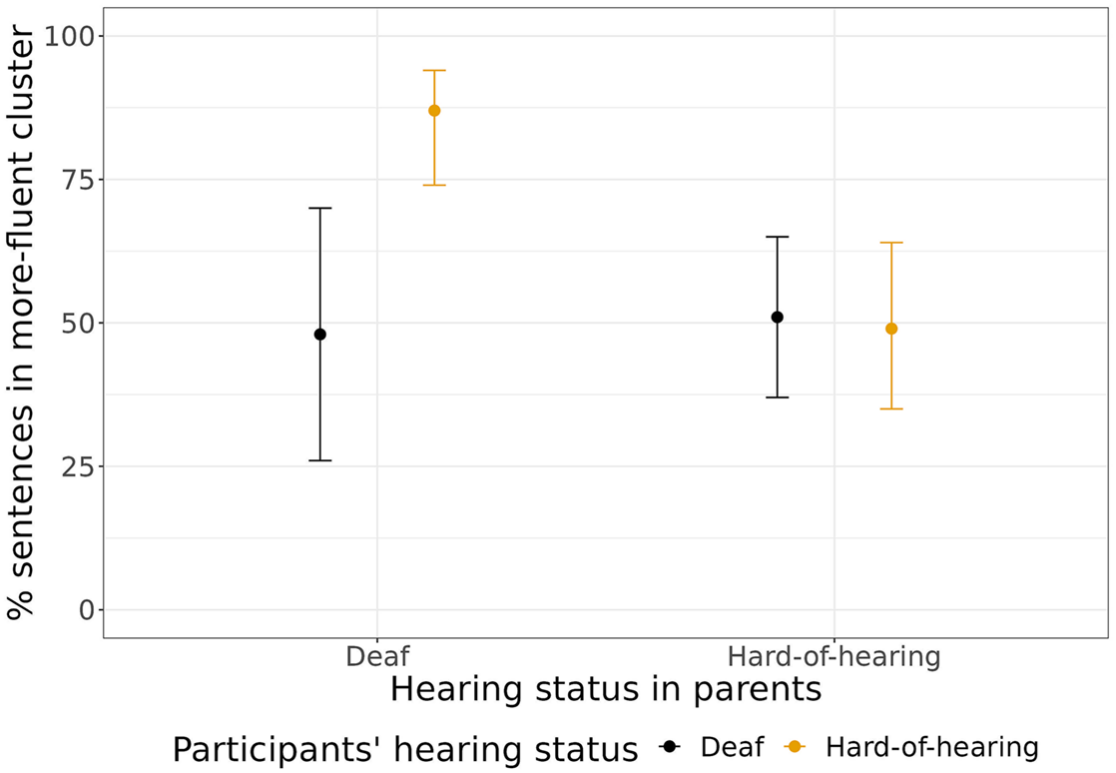
The estimated probability of placement to the more fluent cluster depending on the degree of hearing loss and parents’ hearing status. Error bars represent 95% confidence intervals.

We now turn to the main question of the study, namely whether parents’ hearing status and participants’ degree of hearing loss affect the reading patterns of DHH signers. Mixed-effect model demonstrated that the participant’s degree of hearing loss did not affect cluster membership, whereas parents’ hearing status did, and these two factors interacted (see Table 3; Figure 3): reading patterns of hard-of-hearing children of deaf parents were more likely (estimated 87% probability) to belong to the more fluent cluster than those of hard-of-hearing children of hearing parents (estimated 52% probability) or deaf children of deaf parents (estimated 49% probability). In addition, greater vocabulary size was strongly associated with placement to the more fluent cluster. Gender, age, and the age at which participants started to learn RSL did not affect the probability of cluster placement.

**Table 3:** Parameter estimates for the generalized mixed-effects model for the cluster distribution.

Predictors	Estimate (Log-Odds)	95% CI	*p*-value
(Intercept)	0.10	−0.80–1.01	0.822
Parents’ hearing status (deaf)	1.88	0.54–3.22	**0.012**
Degree of hearing loss (profound)	0.07	−0.95–1.10	0.889
Vocabulary size	0.74	0.42–1.07	**<0.001**
Gender (female)	0.13	−0.20–0.47	0.435
Age	0.03	−0.31–0.37	0.863
Age of Start of RSL usage	−0.09	−0.52–0.34	0.691
Parents’ hearing status × degree of hearing loss	−2.03	−3.43– −0.62	**0.010**
*Random effects*			
σ2	3.29		
τ00 item.id	2.01		
τ00 participant.id	0.82		
τ11 item.id.DeafParents:Deaf	10.95		
τ11 item.id.Deaf	1.96		
τ11 item.id.DeafParents	10.33		
N participants	40		
N item.id	144		
Observations	155,448		
Marginal *R*^2^/Conditional *R*^2^	0.125/0.656		

*p-values in the table are multiplied by two because we adjusted the alpha level by the factor of two. The reason for the adjustment is that we have performed the analysis after having collected data from 37 participants and then decided to proceed with data collection to have data from at least 40 participants.Intercept corresponds to the baseline probability of placement to the more fluent cluster.Bold values indicate statistical significance.

## 5. Discussion

The present study aimed to find out whether early exposure to sign and/or spoken language affects reading fluency in deaf and, especially, hard-of-hearing signers. While early access to sign language has been shown to benefit the reading skills of deaf individuals, it is less clear what role early access to spoken and/or sign language plays for hard-of-hearing individuals with partial access to speech sounds.

Our results suggest a unique advantage for the hard-of-hearing individuals from having early access to both sign and spoken language: native bilingual signers with access to speech sounds were much more likely to have more fluent reading patterns than any other group of participants. Early access to spoken language in hard-of-hearing signers with hearing parents did not correlate with reading fluency. Our results partially support the conclusions of Clark et al. (2016) who claimed that it is early sign language acquisition that is important for later reading fluency. However, early access to sign language seems to affect different groups of participants differentially: participants with partial access to speech sounds benefit from it the most in terms of reading. It seems that hard-of-hearing children born to deaf parents can have the best of both worlds: early access to sign language ensures timely language development, and on top of that, partial access to speech sounds further helps in mastering the spoken and print language system and vocabulary. Our results support the existing claims that early exposure to sign language is beneficial not only for deaf but also for hard-of-hearing children from infancy on (Mayberry, 2007; Freel et al., 2011; Humphries et al., 2014; Hall et al., 2019), and are broadly compatible with claims that bimodal education is effective for proficiency in written language (Lange et al., 2013; Henner et al., 2015).

The role of early access to spoken language is less clear: the lack of significant association between reading fluency and early access to spoken language does not mean that no link between the two exists. Conducting a follow-up study exclusively focused on investigating the impact of early access to spoken language on reading fluency in hard-of-hearing and deaf adult non-signers would provide valuable insights into this debate. However, the results of the current study suggest that for bilingual signers individuals access to spoken language may play a relatively smaller role in reading fluency compared to early access to sign language.

## 6. Conclusion

The current study aimed to evaluate whether and to what degree early access to sign language and early access to spoken language affect reading fluency in adult signers. We found that hard-of-hearing signers with early access to sign language and partial access to spoken language read more fluently than those who were exposed to sign language later in life. No association between early access to spoken language and reading fluency was found. If future studies confirm the greater role of early access to sign language for reading proficiency in hard-of-hearing signers, this could have deep impact on the social and educational policies ensuring the well-being of DHH individuals.

## Data availability statement

The datasets presented in this study can be found in online repositories. The names of the repository/repositories and accession number(s) can be found at: https://osf.io/je8du/.

## Ethics statement

The studies involving humans were approved by the HSE Committee on Interuniversity Surveys and Ethical Assessment of Empirical Research. The studies were conducted in accordance with the local legislation and institutional requirements. The participants provided their written informed consent to participate in this study.

## Author contributions

AZ and AL contributed to conception and design of the study. AZ programmed the procedure and performed data collection and wrote the first draft of the manuscript. OP, AZ, and AL performed the statistical analysis. AL wrote the revised version of the manuscript. All authors contributed to manuscript revision, read, and approved the submitted version.

## Funding

This article is an output of a research project implemented as part of the Basic Research Program at the National Research University Higher School of Economics (HSE University). AL was funded by the Deutsche Forschungsgemeinschaft (DFG, German Research Foundation), project number 317633480, SFB 1287. Funded by the Deutsche Forschungsgemeinschaft (DFG, German Research Foundation) – Projektnummer 491466077.

## Conflict of interest

The authors declare that the research was conducted in the absence of any commercial or financial relationships that could be construed as a potential conflict of interest.

## Publisher’s note

All claims expressed in this article are solely those of the authors and do not necessarily represent those of their affiliated organizations, or those of the publisher, the editors and the reviewers. Any product that may be evaluated in this article, or claim that may be made by its manufacturer, is not guaranteed or endorsed by the publisher.

## Footnotes

^1^Only subjective assessment of RSL proficiency (How proficient would you say you are in RSL on a scale from 1 to 10?) is available because there is no standardized proficiency test for RSL. Self-reported proficiency strongly correlates with objective proficiency measures (Shameem, 1998; Marian et al., 2007).
