# Supplementary material for: Does early exposure to spoken and sign language affect reading fluency in deaf and hard-of-hearing adult signers?
Source: Front Psychol. 2023 Sep 20;14:1145638. doi: 10.3389/fpsyg.2023.1145638 (PMC10548548; doi:10.3389/fpsyg.2023.1145638)
Supplement: Supplementary file 1 [file Table_1.docx]

Supplementary Material

Does early exposure to spoken and sign language affect reading fluency in deaf and hard of hearing adults?

Anastasia A. Ziubanova, Anna K. Laurinavichyute^*^, Olga Parshina

*** Correspondence:** A. Laurinavichyute, anna.laurinavichyute@uni-potsdam.de

# Supplementary Tables

**Supplementary Table 1.** The individual characteristics of each participant. In the hearing loss classification, the degrees of hearing loss correspond to the decibel hearing level given in Table 5 and are based on individual participants’ audiometry results.

| **ID** | **Age** | **Sex** | **RSL proficiency (self-reported) on a scale from 1 to 10** | **Cause** | **Hearing loss** | **Education**  **(in years)** | | **Approx. vocabulary size**  **(in thousands of words)** |
| --- | --- | --- | --- | --- | --- | --- | --- | --- |
| 1 | 23 | female | 10 | other | Severe | 16 | 62000 | |
| 2 | 22 | male | 9 | hereditary | Moderate | 15 | 76000 | |
| 3 | 18 | male | 7 | hereditary | Slight/mild | 12 | 26000 | |
| 4 | 20 | female | 8 | hereditary | Moderately severe | 14 | 39000 | |
| 5 | 21 | male | 9 | other | Severe | 13 | 66000 | |
| 6 | 21 | male | 6 | other | Moderate | 17 | 67000 | |
| 7 | 18 | male | 8 | hereditary | Moderately severe | 13 | 48000 | |
| 8 | 26 | male | 10 | other | Severe | 17 | 44000 | |
| 9 | 39 | female | 10 | hereditary | Severe | 15 | 57000 | |
| 10 | 20 | female | 10 | other | Profound | 13 | 17000 | |
| 11 | 25 | female | 10 | other | Profound | 19 | 34000 | |
| 12 | 38 | female | 10 | other | Severe | 9 | 26000 | |
| 13 | 19 | female | 10 | hereditary | Moderately severe | 13 | 47000 | |
| 14 | 60 | female | 10 | hereditary | Moderate | 11 | 35000 | |
| 15 | 20 | female | 8 | other | Moderately severe | 12 | 71000 | |
| 16 | 34 | female | 10 | hereditary | Profound | 13 | 64000 | |
| 17 | 32 | female | 10 | other | Slight/mild | 17 | 76000 | |
| 18 | 36 | female | 10 | hereditary | Profound | 15 | 35000 | |
| 19 | 38 | female | 10 | other | Severe | 17 | 19000 | |
| 20 | 33 | male | 10 | hereditary | Severe | 18 | 14000 | |
| 21 | 25 | male | 8 | other | Moderately severe | 18 | 24000 | |
| 22 | 24 | male | 10 | other | Severe | 19 | 13000 | |
| 23 | 20 | male | 7 | hereditary | Severe | 14 | 82000 | |
| 24 | 26 | male | 6 | other | Moderate | 16 | 70000 | |
| 25 | 21 | male | 6 | other | Moderately severe | 15 | 46000 | |
| 26 | 24 | male | 5 | other | Profound | 18 | 15000 | |
| 27 | 40 | female | 10 | hereditary | Severe | 17 | 104000 | |
| 28 | 27 | male | 10 | hereditary | Profound | 13 | 18000 | |
| 29 | 27 | male | 10 | hereditary | Profound | 21 | 13000 | |
| 30 | 25 | female | 10 | other | Moderately severe | 20 | 29000 | |
| 31 | 22 | male | 9 | hereditary | Profound | 16 | 13000 | |
| 32 | 26 | female | 9 | hereditary | Profound | 19 | 29000 | |
| 35 | 40 | female | 10 | other | Severe | 20 | 103000 | |
| 36 | 26 | female | 10 | other | Severe | 20 | 18000 | |
| 37 | 38 | female | 10 | hereditary | Severe | 15 | 18000 | |
| 38 | 34 | female | 7 | other | Moderately severe | 17 | 61000 | |
| 39 | 34 | female | 10 | hereditary | Severe | 17 | 49000 | |
| 40 | 32 | female | 9 | hereditary | Profound | 21 | 78000 | |
| 41 | 58 | female | 10 | hereditary | Profound | 18 | 95000 | |
| 42 | 42 | female | 8 | other | Severe | 21 | 52000 | |

**Supplementary Table 2.** Loss range in dB for each degree of hearing loss (Clark, 1981).

| **Degree of hearing loss** | **Hearing loss range**  **(decibel hearing level)** |
| --- | --- |
| Normal | 10 to 15 |
| Slight | 16 to 25 |
| Mild | 26 to 40 |
| Moderate | 41 to 55 |
| Moderately severe | 56 to 70 |
| Severe | 71 to 90 |
| Profound | 91+ |
